# IgG4-Related Disease of the Ovary

**DOI:** 10.5146/tjpath.2020.01500

**Published:** 2021-01-15

**Authors:** Sevda Akyol, Fatma Öz Atalay, Secil Hasdemir, Ömer Yerci

**Affiliations:** Department of Pathology, Bursa Uludag University Faculty of Medicine, Bursa, Turkey; Department of Pathology, Bursa Uludag University Faculty of Medicine, BURSA, TURKEY

**Keywords:** IgG4-related disease, Ovary, Fibrosis, Lymphoplasmacytic infiltration

## Abstract

Immunoglobulin G4-related disease is characterized by dense fibrosis, obliterative phlebitis, and lymphoplasmacytic infiltration that contains abundant IgG4 positive plasma cells. It causes tumefactive lesions in the involved organs and is most commonly seen in the salivary glands, pancreas, and retroperitoneum. Ovarian involvement has been reported in only two cases. In our case, a 58-year-old female patient presented with abdominal distention and pain. Pelvic computed tomography revealed a soft tissue lesion compatible with the omental cake, several intraabdominal implants, and bilateral adnexal fullness. A laparotomy was performed under suspicion of peritoneal carcinomatosis secondary to bilateral adnexal mass. In the histopathologic examination, abundant lymphoplasmacytic infiltration and dense fibrosis were observed in both ovaries and the peritoneum. In the areas of greatest density, the density of IgG4-positive plasma cells was found to range from 40 to 50 per high-power field. The patient was accepted as suffering from probable IgG4-related disease because of the bilateral involvement of the ovaries and the histopathological findings. In conclusion, we present this case to draw attention to the fact that IgG4-related disease can also be seen in the ovary.

## INTRODUCTION

Immunoglobulin G4-related disease (IgG4-RD) is characterized by dense fibrosis, obliterative phlebitis and lymphoplasmacytic infiltration that contains abundant IgG4-positive plasma cells (1). IgG4-RD was first identified in the pancreas as autoimmune pancreatitis (2). Later, lesions similar to those of the pancreas were defined in many organs, including the bile ducts, gallbladder, lymph nodes, retroperitoneum, mesentery, breasts, prostate gland, and skin (3–8). However, IgG4-RD in the female genital tract has been reported in very few cases (9–11).

In this case report, we present probable IgG4-RD involving the bilateral ovaries in which a bilateral ovarian mass together with peritoneal and omental involvement mimicked advanced ovarian cancer.

## CASE REPORT

**Figure 1:**
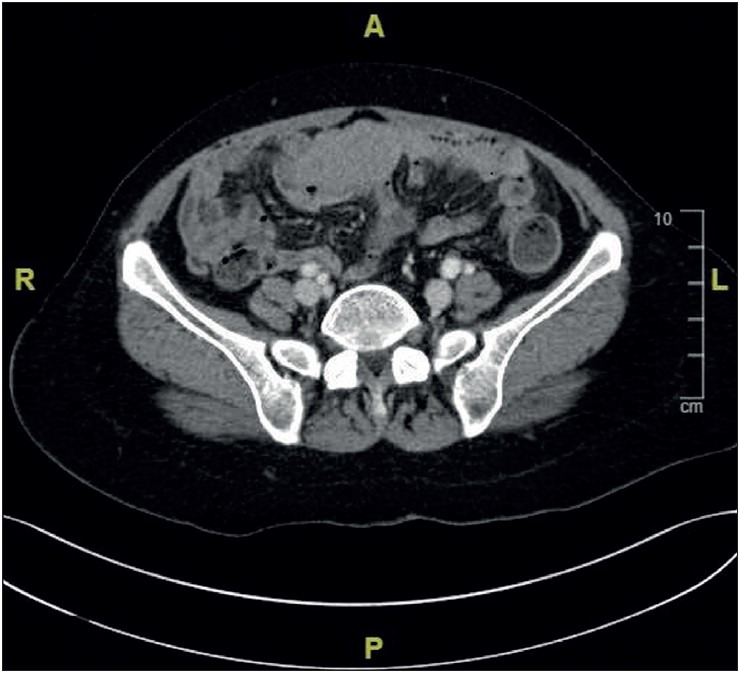
Axial computed tomography images of the pelvis.

A 58-year-old female patient presented with abdominal distention and pain and underwent further investigations after a pelvic mass was detected at an external center. Ultrasonography (USG) revealed mass lesions on both ovaries; the mass on the right ovary was 4 × 3.5 cm, and the mass on the left ovary was 4.2 × 2.7 cm. The largest metastatic nodule in the omentum was 5.4 × 4.2 cm. Pelvic computed tomography (CT) showed a 15-cm long, 3-cm thick soft tissue lesion compatible with the omental cake, several intraabdominal implants suggesting increases in nodular thickness, bilateral adnexal fullness, and pelvic peritoneal implants (Figure 1). As a result of these radiological findings, peritoneal carcinomatosis was considered, and the patient underwent hysterectomy and bilateral salpingo-oophorectomy due to the adnexal mass. The patient’s blood values were normal, and only CA125 was high (72 U/mL) among the tumor markers.

When the tissues were examined in frozen section, it was observed that the lesion contained intense fibrosis. Disseminated peritoneal leiomyomatosis was considered in the differential diagnosis due to the diffuse peritoneal involvement. Although a benign lesion was considered first, the final diagnosis was left to examination of the permanent sections.

Macroscopic examination of the specimen showed areas of gray-white discoloration, the largest of which was 3 × 2.5 cm in diameter, on the anterior-posterior wall of the uterus and around the right and left tuba uterina. The right ovary was 3.5 cm in diameter, and the left ovary was 4 cm in diameter; the cross-sectional surfaces were irregular. Also, multiple intraabdominal serosal implants were located on the intestine and the appendix and in the right and left paracolic regions.

**Figure 2:**
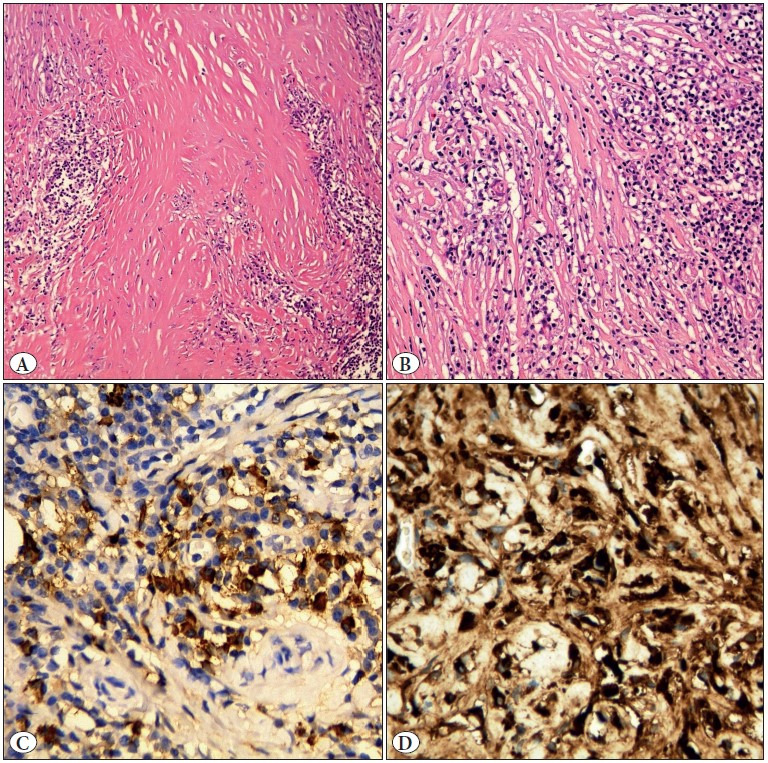
**A)** Dense fibrosis (H&E; x400). **B)** A dense parenchymal lymphoplasmacytic inflammatory infiltrate (H&E; x400). **C)** Immunohistochemical study showing the density of IgG4 positive cells. It ranged from 40 to 50 IgG4 positive cells per HPF (IHC; x400). **D)** Immunohistochemical study showing the density of IgG positive cells (IHC; x400).

On microscopic examination, intense fibrosis in an occasional storiform pattern was seen in both ovaries and peritoneal implants. Significant lymphoid follicles and intense lymphoplasmacytic inflammatory cell infiltration on the fibrotic ground were noteworthy. An immunohistochemical study determined the density of IgG4-positive cells. In a high power field (HPF), 40 to 50 IgG4 positive cells were found in the area of greatest density. The IgG4+/ IgG+ cell ratio was more than 40% (Figure 2). In conclusion, the patient was accepted as probable with IgG4-RD because of the bilateral involvement of the ovaries and the histopathological findings.

Blood samples taken for IgG1, IgG2, IgG3 and IgG4 were within normal limits three weeks after the patient’s operation. No additional treatment was given to the patient following the operation.

## DISCUSSION

IgG4-RD is a relatively recently defined systemic fibroinflammatory process (12). It was first reported in the pancreas in 2001 (2). Since then, it has been identified in many organs in the body.

When diagnosing IgG4-RD, clinical, serological, and histopathological features are evaluated. IgG4-RD can affect a single organ or multiple organ systems and can be found bilaterally in organs, and an increase in the size of the affected organs or impairment of organ functions occurs (13). Among the serological findings, the serum IgG4 concentration is expected to be >135 mg/dl; however, the serum IgG4 level did not increase in half of the reported cases (14). Besides, the serum IgG4 level may also be high in patients with chronic diseases and certain malignancies (15). The major histopathological findings of IgG4-RD include lymphoplasmacytic infiltration, storiform fibrosis and obliterative phlebitis. Lymphoplasmacytic infiltration is rich in IgG4 + plasma cells and often eosinophil leukocytes (12). More than 10 IgG4 positive cells are present in the HPF of the biopsy samples, and the IgG4+/IgG+ cell ratio is more than 40%.

If these three criteria are met, the diagnosis of IgG4-RD is definitive. Patients who meet only one of the serological or histopathological criteria and have organ symptoms probably have IgG4-RD. Patients with organ symptoms who do not meet the serological or histopathological criteria are unlikely to have IgG4-RD (13).

There are very few cases of IgG4-RD with ovarian involvement in the literature. In our case, bilateral ovarian enlargement was accompanied by peritoneal and omental implants as in the case reported by Maruyama (10). In a case reported by Sekulic, the ovarian involvement was unilateral (11). With these clinical findings, the preliminary diagnosis was advanced ovarian malignancy in both our case and Maruyama’s case (10).

In our case, IgG4 levels were within normal limits in the blood samples taken three weeks postoperatively. In Maruyama’s reported case, serum IgG4 concentration was 1,000 mg/dL on day seven postoperatively, and in Sekulic’s reported case, serum IgG4 concentration was not determined (10,11).

Histopathologically, intense lymphoplasmacytic infiltra-tion was observed in all three ovarian IgG4-RD cases. In Sekulic’s case, the inflammation was reported to be rich in eosinophils (11). In our case, there was intense fibrosis, whereas only Maruyama among the other two cases emphasized fibrosis (10). We did not find phlebitis in our case, but phlebitis was seen in the other two cases (10,11). The number of IgG4-positive cells required for the diagnosis of IgG4-RD varies with the organ involved (12). In Maruyama’s reported case, more than 90% of the plasma cells in the HPF showed positive staining immunohistochemically with IgG4 (10). In Sekulic’s reported case, immunohistochemically 40 to 50 IgG4-positive cells were found per HPF, similar to our case (11).

In terms of histopathological features, multiple myeloma, lymphoproliferative diseases, spindle cell mesenchymal neoplasms, multicentric Castleman disease, and infections should be considered in the differential diagnosis of IgG4-RD. Therefore, when examining a sample with IgG4-RD-like histology, its clinical history should be considered. The evaluation of IgG4-RD in frozen section may also be difficult due to pronounced fibrosis. Disseminated peritoneal leiomyomatosis can be considered in the differential diagnosis in frozen evaluation.

In our case, luteinized thecoma associated with sclerosing peritonitis was also included in the differential diagnosis due to the bilateral ovarian and peritoneal involvement. However, the absence of luteinized cells and the presence of intense lymphoplasmacytic inflammation with storiform fibrosis ruled out this disease.

In conclusion, we reported the third case of ovarian IgG4-RD. In histopathological examinations, IgG4-RD, although very rare, should be considered in the differential diagnosis, particularly if marked lymphoplasmacytic inflammation with storiform fibrosis is observed in ovary. IgG4-RD responds well to prednisone therapy, which increases the importance of a correct diagnosis. Correct diagnosis also prevents unnecessary surgical interventions.

## CONFLICT of INTEREST

The authors declare no conflict of interest.
